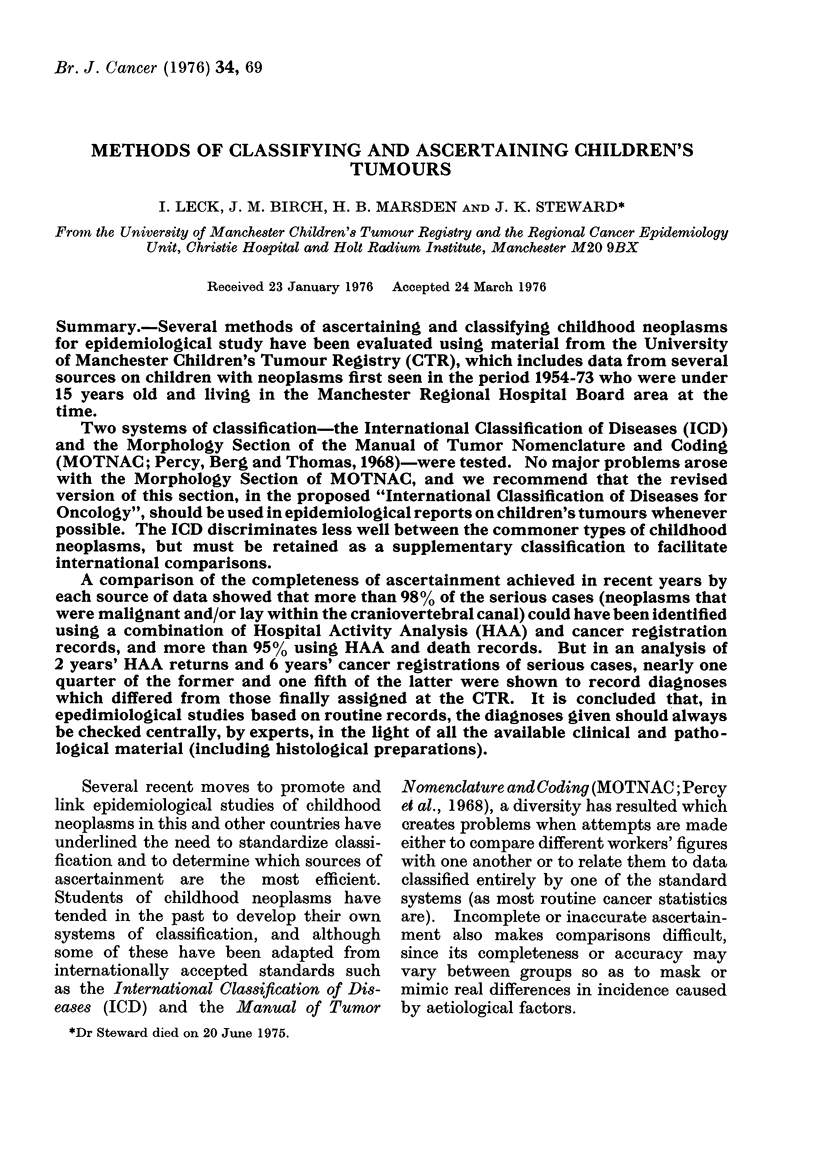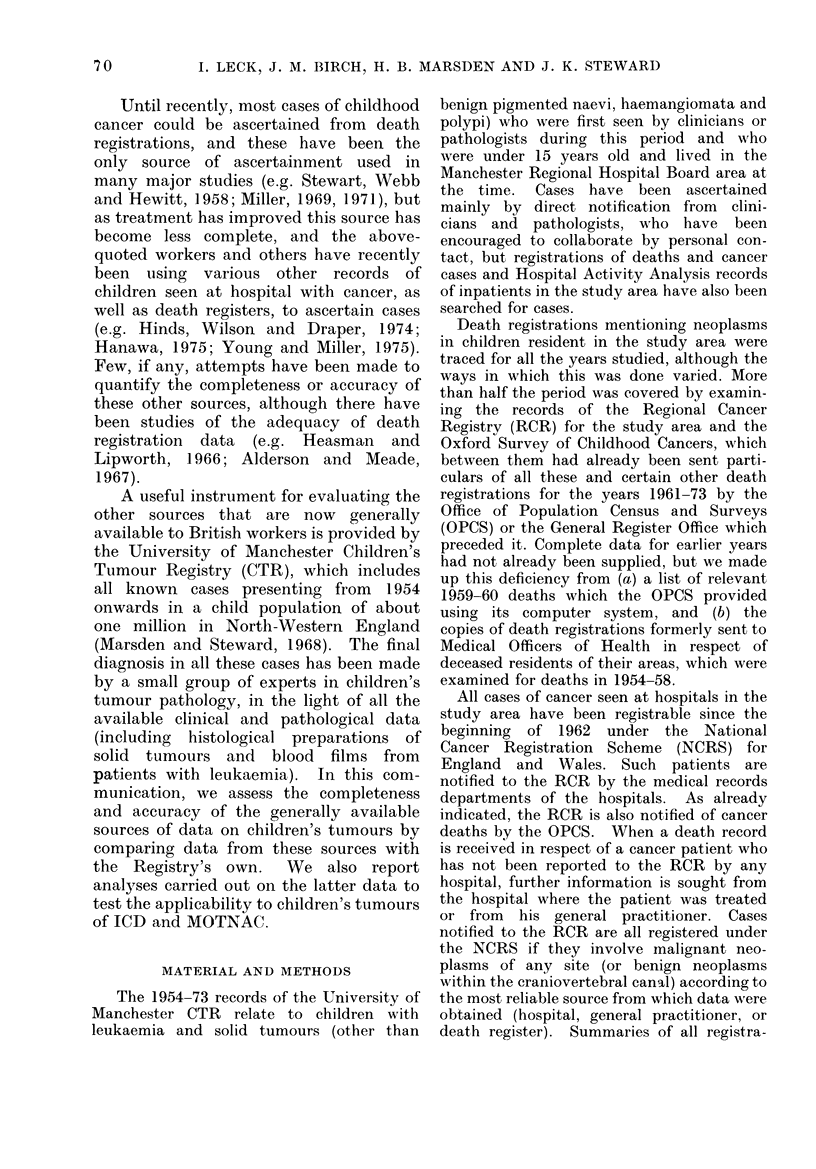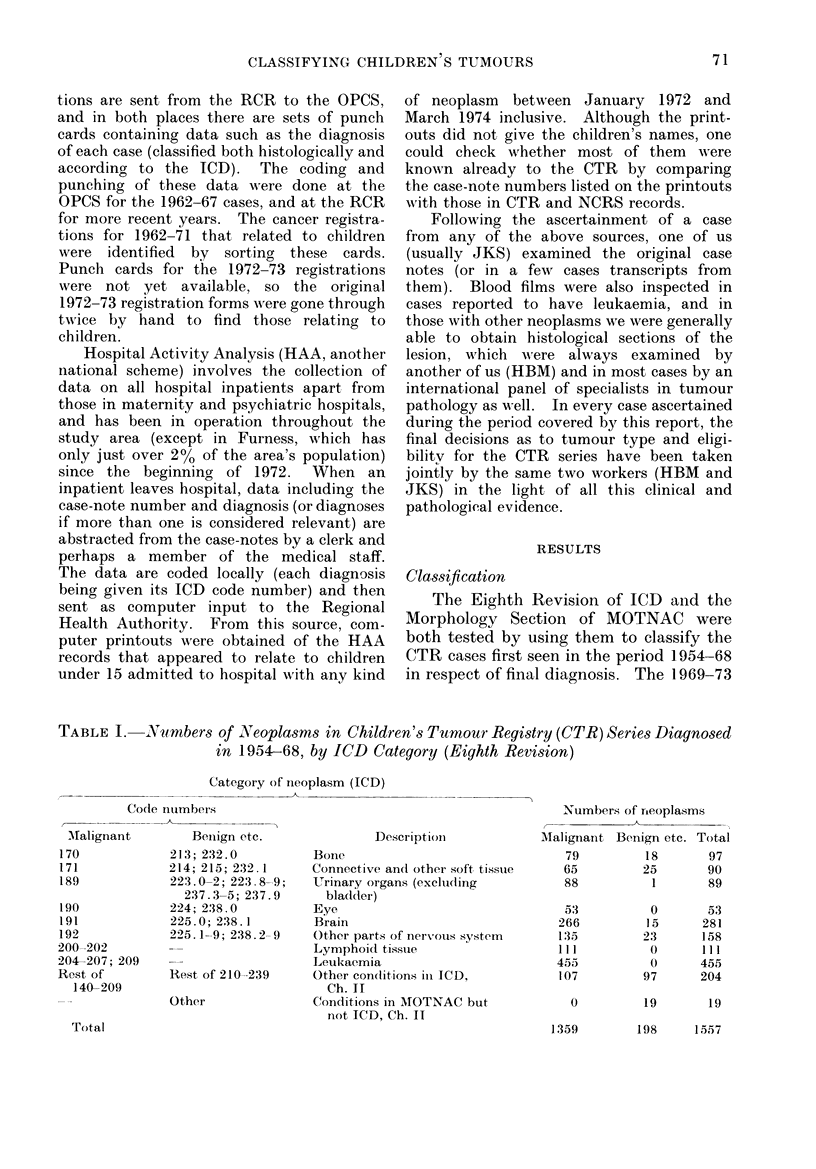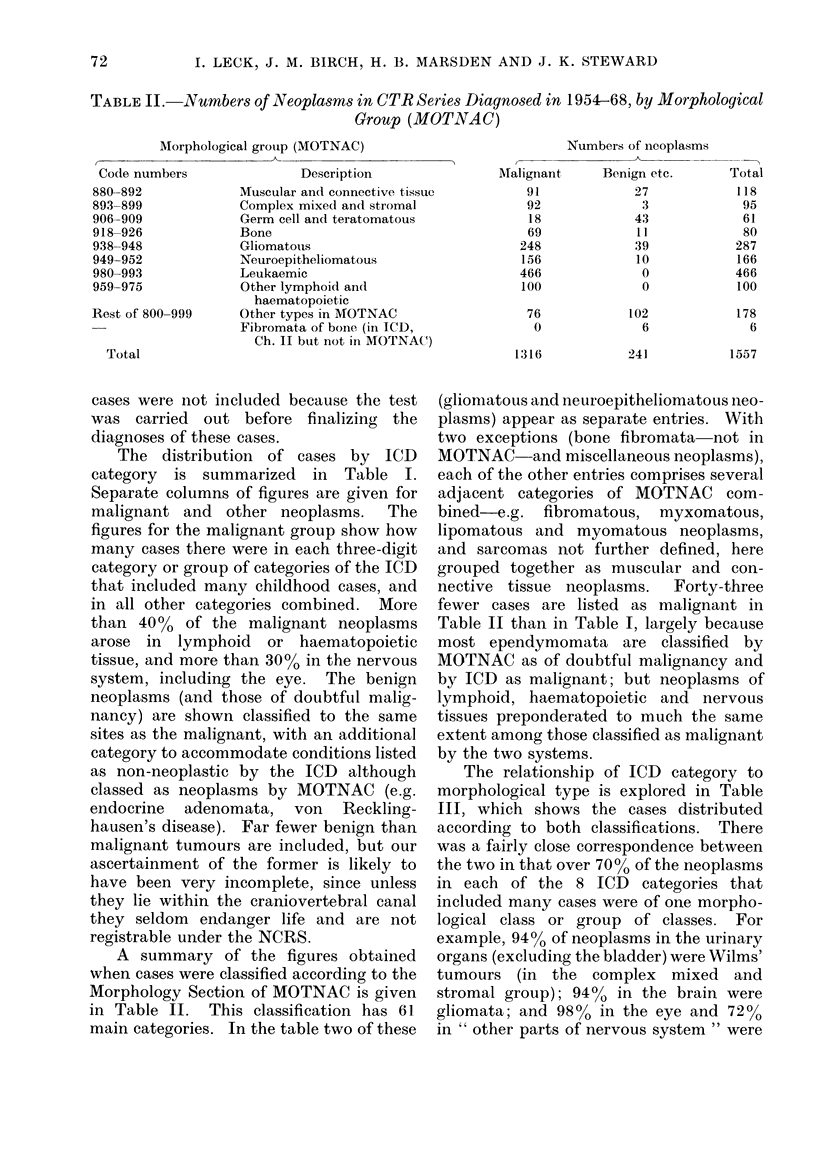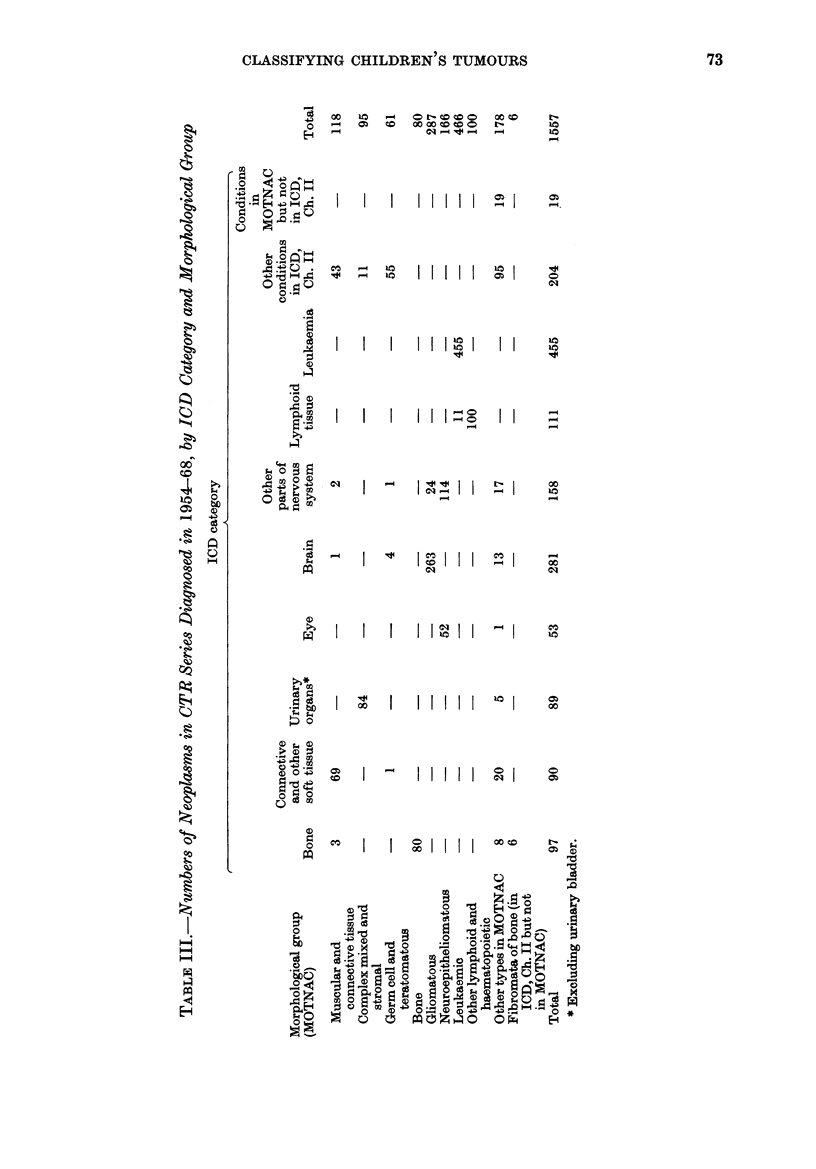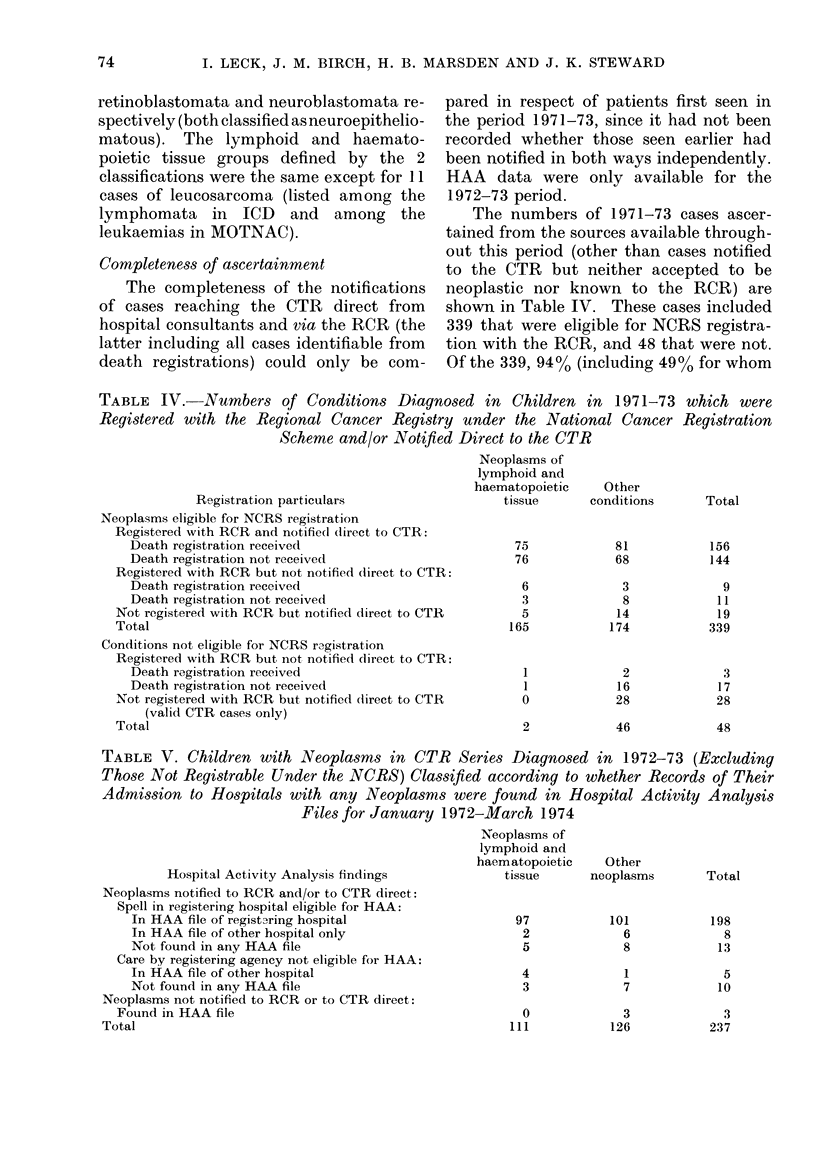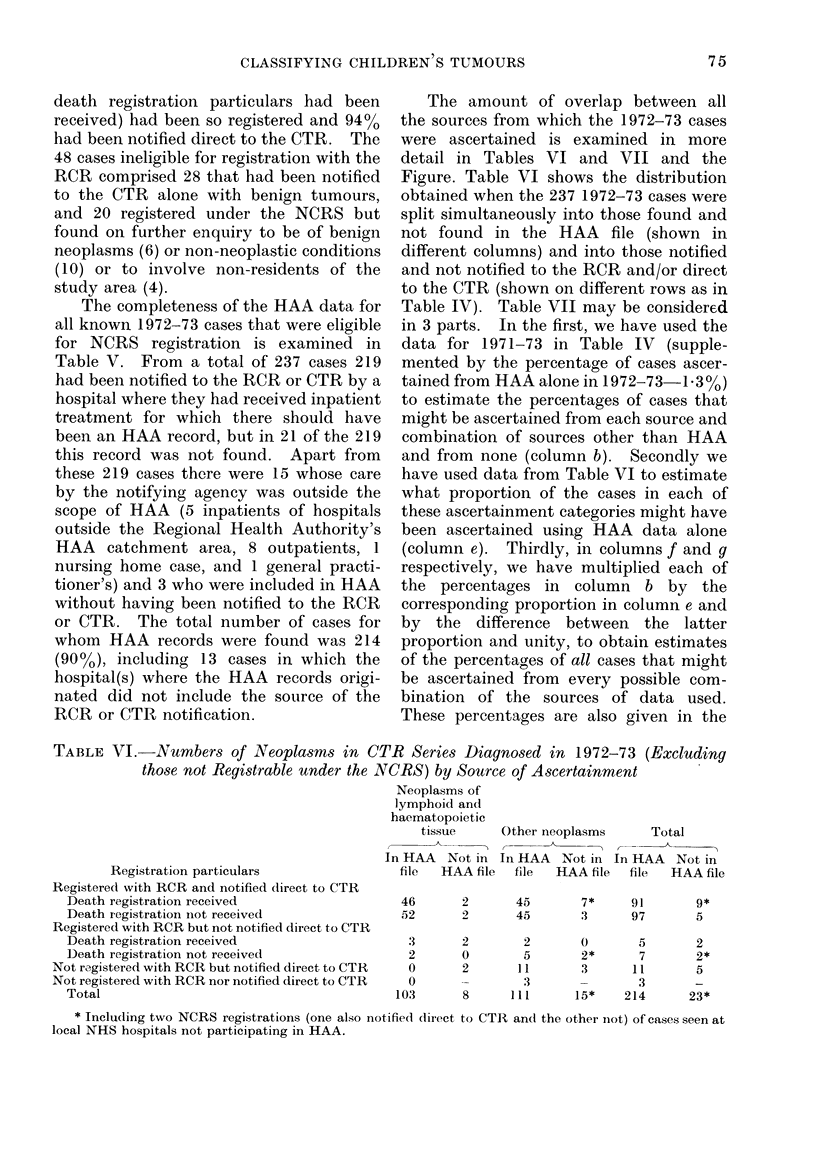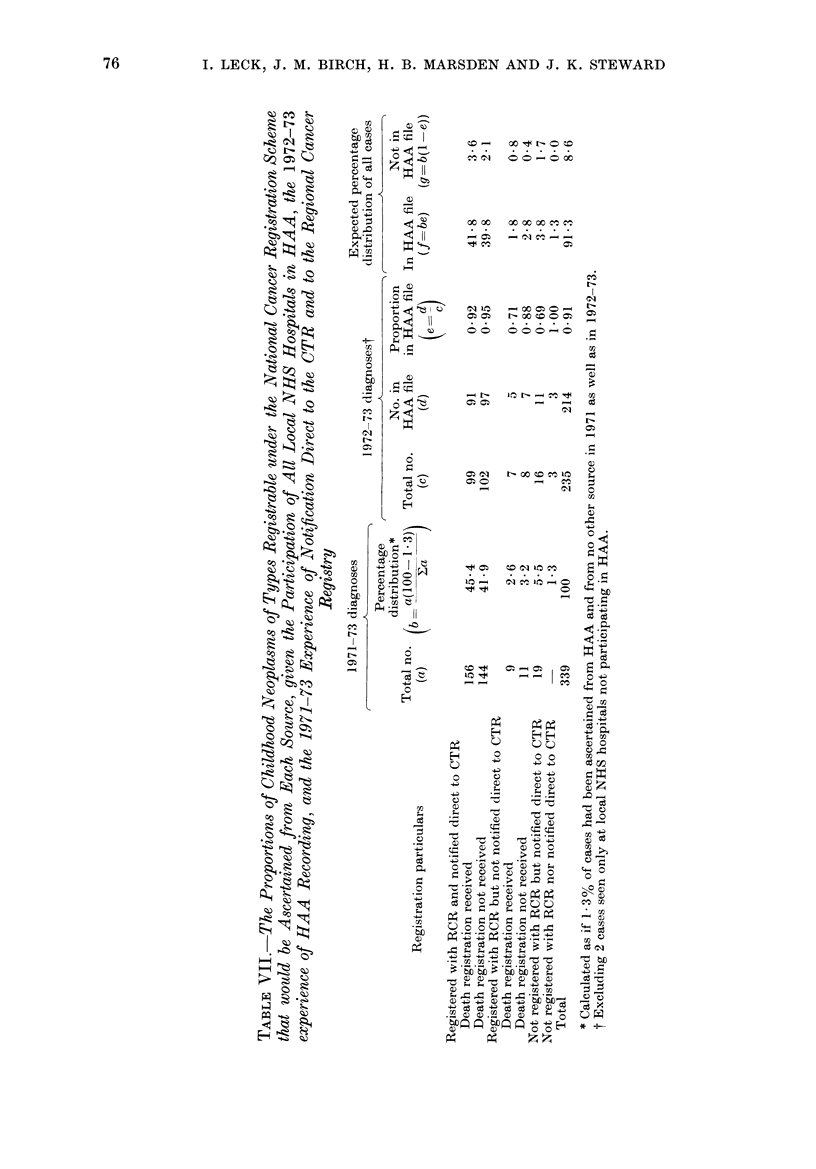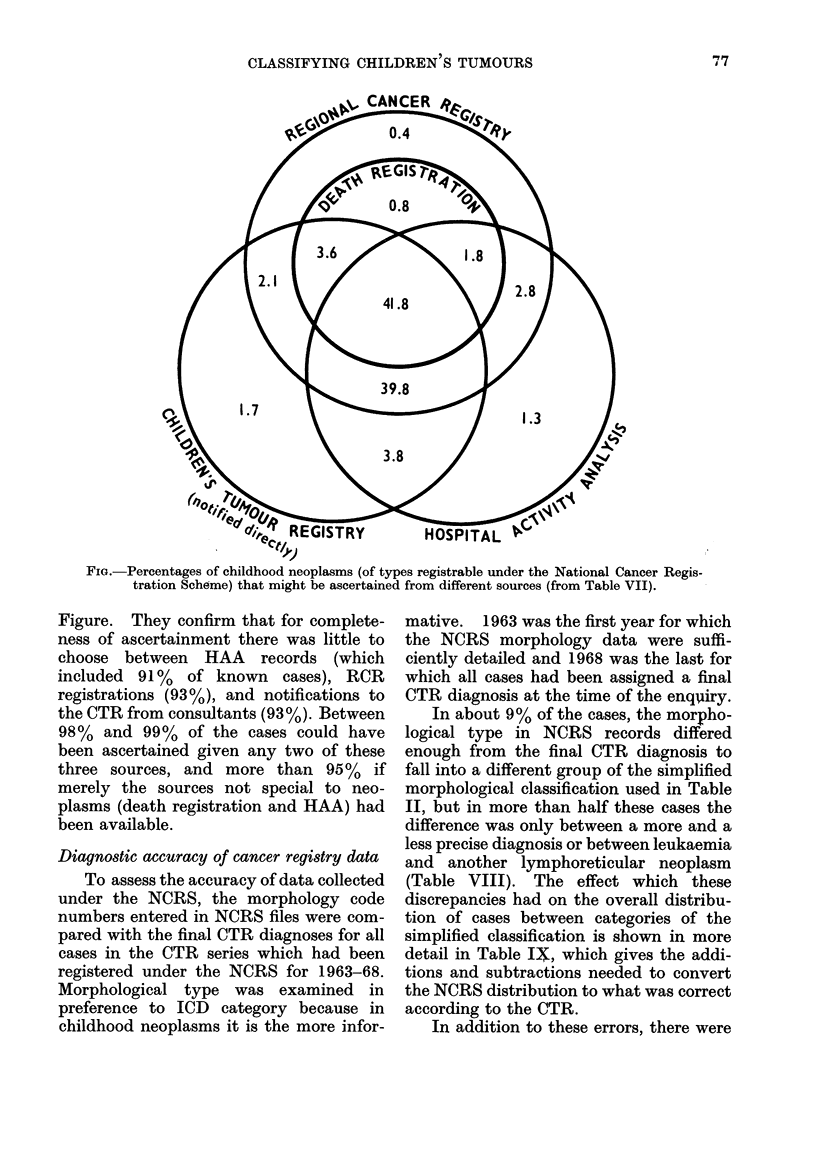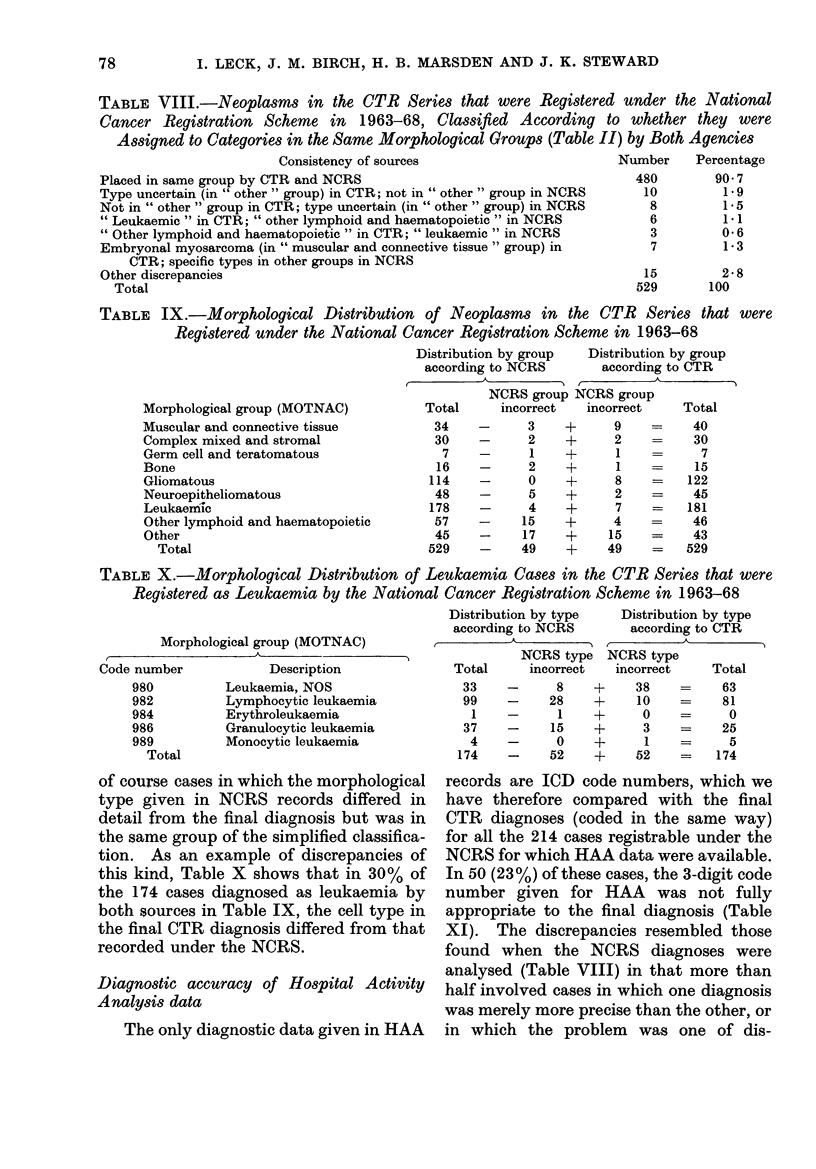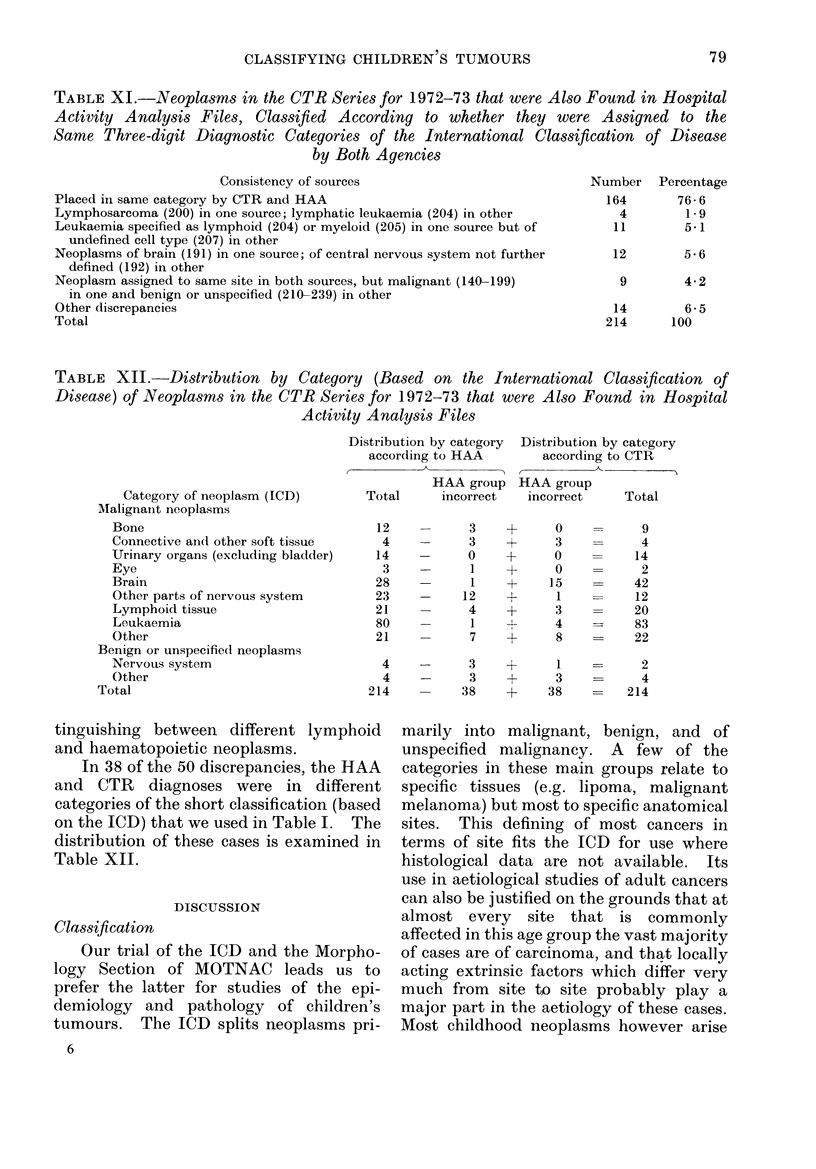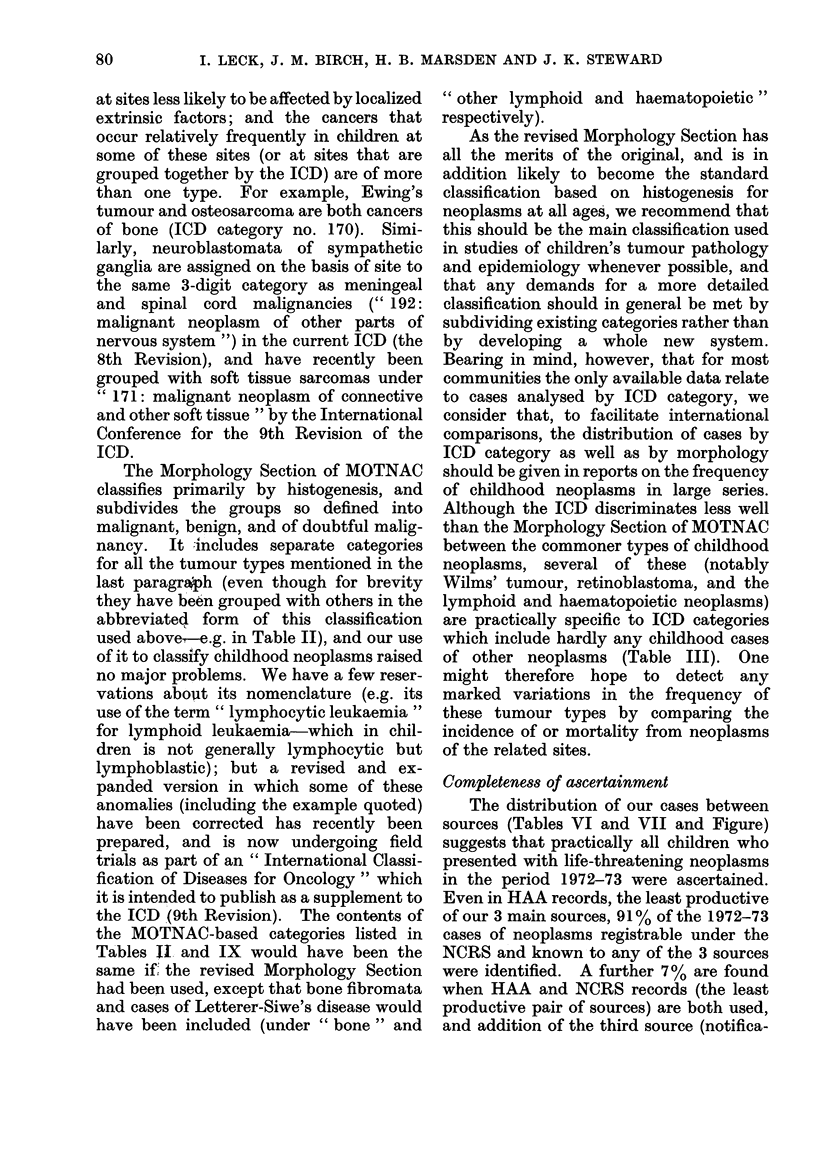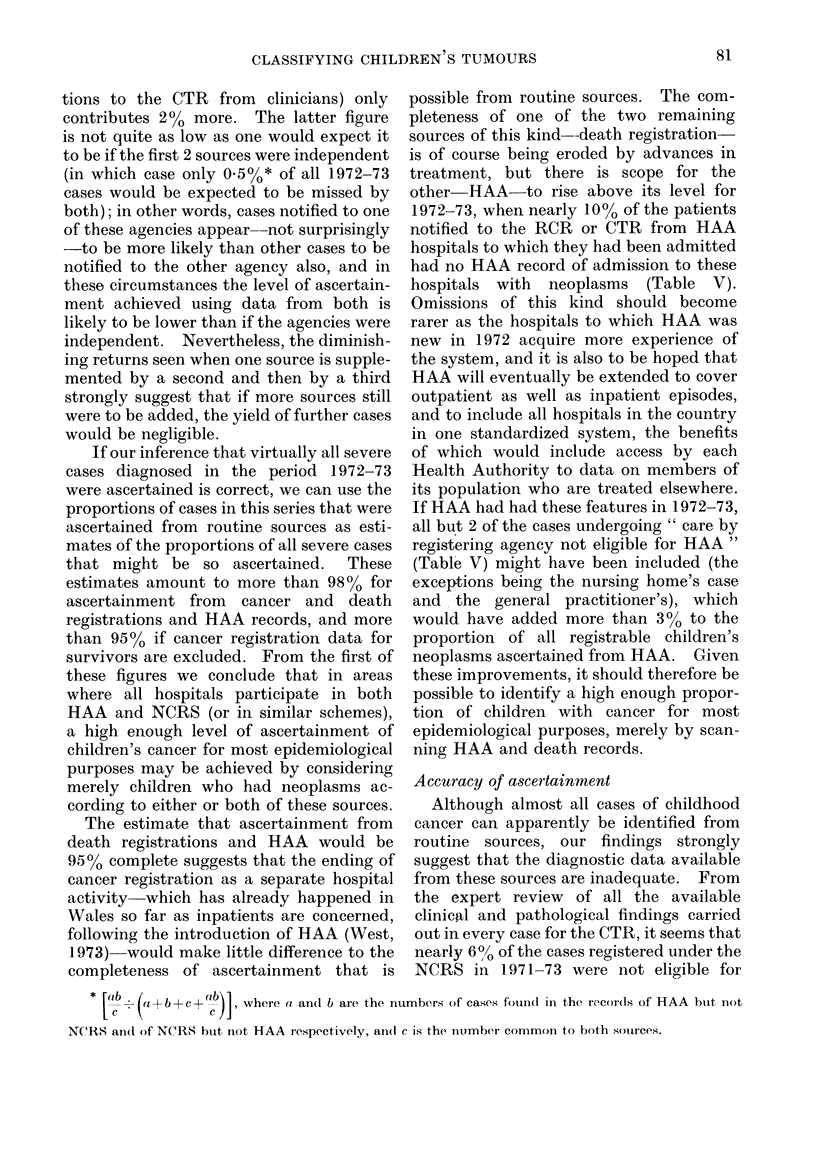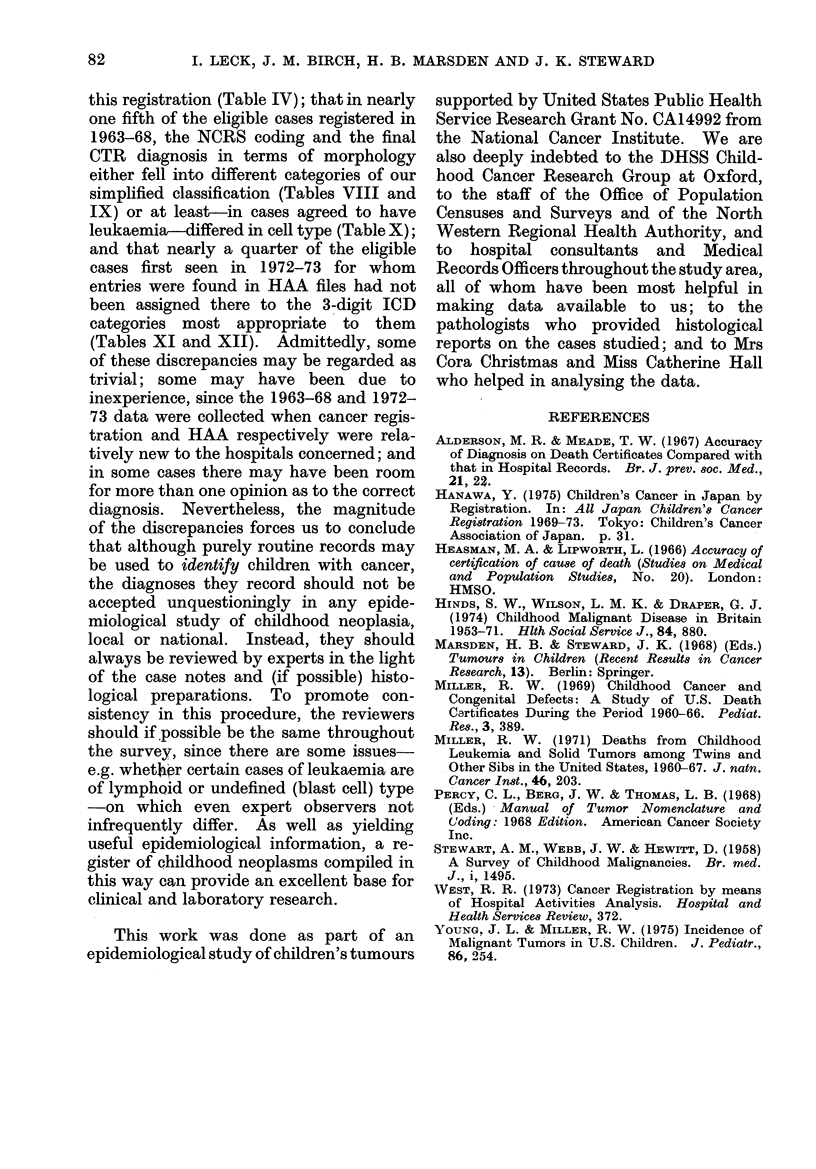# Methods of classifying and ascertaining children's tumours.

**DOI:** 10.1038/bjc.1976.124

**Published:** 1976-07

**Authors:** I. Leck, J. M. Birch, H. B. Marsden, J. K. Steward

## Abstract

Several methods of ascertaining and classifying childhood neoplasms for epidemiological study have been evaluated using material from the University of Manchester Children's Tumour Registry (CTR), which includes data from several sources on children with neoplasms first seen in the period 1954-73 who were under 15 years old and living in the Manchester Regional Hospital Board area at the time. Two systems of classification-the International Classification of Diseases (ICD) and the Morphology Section of the Manual of Tumor Nomenclature and Coding (MOTNAC; Percy, Berg and Thomas, 1968)-were tested. No major problems arose with the Morphology Section of MOTNAC, and we recommend that the revised version of this section, in the proposed "International Classification of Diseases for Oncology", should be used in epidemiological reports on children's tumours whenever possible. The ICD discriminates less well between the commoner types of childhood neoplasms, but must be retained as a supplementary classification to facilitate international comparisons. A comparison of the completeness of ascertainment achieved in recent years by each source of data showed that more than 98% of the serious cases (neoplasms that were malignant and/or lay within the craniovertebral canal) could have been identified using a combination of Hospital Activity Analysis (HAA) and cancer registration records, and more than 95% using HAA and death records. But in an analysis of 2 years' HAA returns and 6 years' cancer registrations of serious cases, nearly one quarter of the former and one fifth of the latter were shown to record diagnoses which differed from those finally assigned at the CTR. It is concluded that, in epedimiological studies based on routine records, the diagnoses given should always be checked centrally, by experts, in the light of all the available clinical and pathological material (including histological preparations).


*Br. J. Cancer* (1976) **34**, 69

### METHODS OF CLASSIFYING AND ASCERTAINING CHILDREN'S TUMOURS

I. LECK, J. M. BIRCH, H. B. MARSDEN AND J. K. STEWARD*

*From the University of Manchester Children's Tumour Registry and the Regional Cancer Epidemiology Unit, Christie Hospital and Holt Radium Institute, Manchester M20 9BX*

Received 23 January 1976 Accepted 24 March 1976

**Summary.—Several methods of ascertaining and classifying childhood neoplasms for epidemiological study have been evaluated using material from the University of Manchester Children's Tumour Registry (CTR), which includes data from several sources on children with neoplasms first seen in the period 1954-73 who were under 15 years old and living in the Manchester Regional Hospital Board area at the time.**

**Two systems of classification—the International Classification of Diseases (ICD) and the Morphology Section of the Manual of Tumor Nomenclature and Coding (MOTNAC; Percy, Berg and Thomas, 1968)—were tested. No major problems arose with the Morphology Section of MOTNAC, and we recommend that the revised version of this section, in the proposed "International Classification of Diseases for Oncology", should be used in epidemiological reports on children's tumours whenever possible. The ICD discriminates less well between the commoner types of childhood neoplasms, but must be retained as a supplementary classification to facilitate international comparisons.**

**A comparison of the completeness of ascertainment achieved in recent years by each source of data showed that more than 98% of the serious cases (neoplasms that were malignant and/or lay within the craniovertebral canal) could have been identified using a combination of Hospital Activity Analysis (HAA) and cancer registration records, and more than 95% using HAA and death records. But in an analysis of 2 years' HAA returns and 6 years' cancer registrations of serious cases, nearly one quarter of the former and one fifth of the latter were shown to record diagnoses which differed from those finally assigned at the CTR. It is concluded that, in epedimiological studies based on routine records, the diagnoses given should always be checked centrally, by experts, in the light of all the available clinical and pathological material (including histological preparations).**

Several recent moves to promote and link epidemiological studies of childhood neoplasms in this and other countries have underlined the need to standardize classification and to determine which sources of ascertainment are the most efficient. Students of childhood neoplasms have tended in the past to develop their own systems of classification, and although some of these have been adapted from internationally accepted standards such as the *International Classification of Diseases* (ICD) and the *Manual of Tumor Nomenclature and Coding* (MOTNAC; Percy *et al.*, 1968), a diversity has resulted which creates problems when attempts are made either to compare different workers' figures with one another or to relate them to data classified entirely by one of the standard systems (as most routine cancer statistics are). Incomplete or inaccurate ascertainment also makes comparisons difficult, since its completeness or accuracy may vary between groups so as to mask or mimic real differences in incidence caused by aetiological factors.

*Dr Steward died on 20 June 1975.

Until recently, most cases of childhood cancer could be ascertained from death registrations, and these have been the only source of ascertainment used in many major studies (e.g. Stewart, Webb and Hewitt, 1958; Miller, 1969, 1971), but as treatment has improved this source has become less complete, and the above-quoted workers and others have recently been using various other records of children seen at hospital with cancer, as well as death registers, to ascertain cases (e.g. Hinds, Wilson and Draper, 1974; Hanawa, 1975; Young and Miller, 1975). Few, if any, attempts have been made to quantify the completeness or accuracy of these other sources, although there have been studies of the adequacy of death registration data (e.g. Heasman and Lipworth, 1966; Alderson and Meade, 1967).

A useful instrument for evaluating the other sources that are now generally available to British workers is provided by the University of Manchester Children's Tumour Registry (CTR), which includes all known cases presenting from 1954 onwards in a child population of about one million in North-Western England (Marsden and Steward, 1968). The final diagnosis in all these cases has been made by a small group of experts in children's tumour pathology, in the light of all the available clinical and pathological data (including histological preparations of solid tumours and blood films from patients with leukaemia). In this communication, we assess the completeness and accuracy of the generally available sources of data on children's tumours by comparing data from these sources with the Registry's own. We also report analyses carried out on the latter data to test the applicability to children's tumours of ICD and MOTNAC.

### MATERIAL AND METHODS

The 1954–73 records of the University of Manchester CTR relate to children with leukaemia and solid tumours (other than benign pigmented naevi, haemangiomata and polypi) who were first seen by clinicians or pathologists during this period and who were under 15 years old and lived in the Manchester Regional Hospital Board area at the time. Cases have been ascertained mainly by direct notification from clinicians and pathologists, who have been encouraged to collaborate by personal contact, but registrations of deaths and cancer cases and Hospital Activity Analysis records of inpatients in the study area have also been searched for cases.

Death registrations mentioning neoplasms in children resident in the study area were traced for all the years studied, although the ways in which this was done varied. More than half the period was covered by examining the records of the Regional Cancer Registry (RCR) for the study area and the Oxford Survey of Childhood Cancers, which between them had already been sent particulars of all these and certain other death registrations for the years 1961–73 by the Office of Population Census and Surveys (OPCS) or the General Register Office which preceded it. Complete data for earlier years had not already been supplied, but we made up this deficiency from (*a*) a list of relevant 1959–60 deaths which the OPCS provided using its computer system, and (*b*) the copies of death registrations formerly sent to Medical Officers of Health in respect of deceased residents of their areas, which were examined for deaths in 1954–58.

All cases of cancer seen at hospitals in the study area have been registrable since the beginning of 1962 under the National Cancer Registration Scheme (NCRS) for England and Wales. Such patients are notified to the RCR by the medical records departments of the hospitals. As already indicated, the RCR is also notified of cancer deaths by the OPCS. When a death record is received in respect of a cancer patient who has not been reported to the RCR by any hospital, further information is sought from the hospital where the patient was treated or from his general practitioner. Cases notified to the RCR are all registered under the NCRS if they involve malignant neoplasms of any site (or benign neoplasms within the craniovertebral canal) according to the most reliable source from which data were obtained (hospital, general practitioner, or death register). Summaries of all registrations are sent from the RCR to the OPCS, and in both places there are sets of punch cards containing data such as the diagnosis of each case (classified both histologically and according to the ICD). The coding and punching of these data were done at the OPCS for the 1962–67 cases, and at the RCR for more recent years. The cancer registrations for 1962–71 that related to children were identified by sorting these cards. Punch cards for the 1972–73 registrations were not yet available, so the original 1972–73 registration forms were gone through twice by hand to find those relating to children.

Hospital Activity Analysis (HAA, another national scheme) involves the collection of data on all hospital inpatients apart from those in maternity and psychiatric hospitals, and has been in operation throughout the study area (except in Furness, which has only just over 2% of the area's population) since the beginning of 1972. When an inpatient leaves hospital, data including the case-note number and diagnosis (or diagnoses if more than one is considered relevant) are abstracted from the case-notes by a clerk and perhaps a member of the medical staff. The data are coded locally (each diagnosis being given its ICD code number) and then sent as computer input to the Regional Health Authority. From this source, computer printouts were obtained of the HAA records that appeared to relate to children under 15 admitted to hospital with any kind of neoplasm between January 1972 and March 1974 inclusive. Although the printouts did not give the children's names, one could check whether most of them were known already to the CTR by comparing the case-note numbers listed on the printouts with those in CTR and NCRS records.

Following the ascertainment of a case from any of the above sources, one of us (usually JKS) examined the original case notes (or in a few cases transcripts from them). Blood films were also inspected in cases reported to have leukaemia, and in those with other neoplasms we were generally able to obtain histological sections of the lesion, which were always examined by another of us (HBM) and in most cases by an international panel of specialists in tumour pathology as well. In every case ascertained during the period covered by this report, the final decisions as to tumour type and eligibility for the CTR series have been taken jointly by the same two workers (HBM and JKS) in the light of all this clinical and pathological evidence.

### RESULTS

### *Classification*

The Eighth Revision of ICD and the Morphology Section of MOTNAC were both tested by using them to classify the CTR cases first seen in the period 1954–68 in respect of final diagnosis. The 1969–73

TABLE I.—*Numbers of Neoplasms in Children's Tumour Registry (CTR) Series Diagnosed in 1954–68, by ICD Category (Eighth Revision)*

| Category of neoplasm (ICD) | | | Numbers of neoplasms | | |
|---|---|---|---|---|---|
| Code numbers | | | | | |
| Malignant | Benign etc. | Description | Malignant | Benign etc. | Total |
| 170 | 213; 232.0 | Bone | 79 | 18 | 97 |
| 171 | 214; 215; 232.1 | Connective and other soft tissue | 65 | 25 | 90 |
| 189 | 223.0–2; 223.8–9; 237.3–5; 237.9 | Urinary organs (excluding bladder) | 88 | 1 | 89 |
| 190 | 224; 238.0 | Eye | 53 | 0 | 53 |
| 191 | 225.0; 238.1 | Brain | 266 | 15 | 281 |
| 192 | 225.1–9; 238.2–9 | Other parts of nervous system | 135 | 23 | 158 |
| 200–202 | — | Lymphoid tissue | 111 | 0 | 111 |
| 204–207; 209 | — | Leukaemia | 455 | 0 | 455 |
| Rest of 140–209 | Rest of 210–239 | Other conditions in ICD, Ch. II | 107 | 97 | 204 |
| — | Other | Conditions in MOTNAC but not ICD, Ch. II | 0 | 19 | 19 |
| Total | | | 1359 | 198 | 1557 |

TABLE II.—*Numbers of Neoplasms in CTR Series Diagnosed in* 1954–68, *by Morphological Group* (*MOTNAC*)

| Morphological group (MOTNAC) | | Numbers of neoplasms | | |
|---|---|---|---|---|
| Code numbers | Description | Malignant | Benign etc. | Total |
| 880–892 | Muscular and connective tissue | 91 | 27 | 118 |
| 893–899 | Complex mixed and stromal | 92 | 3 | 95 |
| 906–909 | Germ cell and teratomatous | 18 | 43 | 61 |
| 918–926 | Bone | 69 | 11 | 80 |
| 938–948 | Gliomatous | 248 | 39 | 287 |
| 949–952 | Neuroepitheliomatous | 156 | 10 | 166 |
| 980–993 | Leukaemic | 466 | 0 | 466 |
| 959–975 | Other lymphoid and haematopoietic | 100 | 0 | 100 |
| Rest of 800–999 | Other types in MOTNAC | 76 | 102 | 178 |
| — | Fibromata of bone (in ICD, Ch. II but not in MOTNAC) | 0 | 6 | 6 |
| Total | | 1316 | 241 | 1557 |

cases were not included because the test was carried out before finalizing the diagnoses of these cases.

The distribution of cases by ICD category is summarized in Table I. Separate columns of figures are given for malignant and other neoplasms. The figures for the malignant group show how many cases there were in each three-digit category or group of categories of the ICD that included many childhood cases, and in all other categories combined. More than 40% of the malignant neoplasms arose in lymphoid or haematopoietic tissue, and more than 30% in the nervous system, including the eye. The benign neoplasms (and those of doubtful malignancy) are shown classified to the same sites as the malignant, with an additional category to accommodate conditions listed as non-neoplastic by the ICD although classed as neoplasms by MOTNAC (e.g. endocrine adenomata, von Recklinghausen's disease). Far fewer benign than malignant tumours are included, but our ascertainment of the former is likely to have been very incomplete, since unless they lie within the craniovertebral canal they seldom endanger life and are not registrable under the NCRS.

A summary of the figures obtained when cases were classified according to the Morphology Section of MOTNAC is given in Table II. This classification has 61 main categories. In the table two of these (gliomatous and neuroepitheliomatous neoplasms) appear as separate entries. With two exceptions (bone fibromata—not in MOTNAC—and miscellaneous neoplasms), each of the other entries comprises several adjacent categories of MOTNAC combined—e.g. fibromatous, myxomatous, lipomatous and myomatous neoplasms, and sarcomas not further defined, here grouped together as muscular and connective tissue neoplasms. Forty-three fewer cases are listed as malignant in Table II than in Table I, largely because most ependymomata are classified by MOTNAC as of doubtful malignancy and by ICD as malignant; but neoplasms of lymphoid, haematopoietic and nervous tissues preponderated to much the same extent among those classified as malignant by the two systems.

The relationship of ICD category to morphological type is explored in Table III, which shows the cases distributed according to both classifications. There was a fairly close correspondence between the two in that over 70% of the neoplasms in each of the 8 ICD categories that included many cases were of one morphological class or group of classes. For example, 94% of neoplasms in the urinary organs (excluding the bladder) were Wilms' tumours (in the complex mixed and stromal group); 94% in the brain were gliomata; and 98% in the eye and 72% in " other parts of nervous system " were

TABLE III.—*Numbers of Neoplasms in CTR Series Diagnosed in* 1954–68, *by ICD Category and Morphological Group*

| Morphological group (MOTNAC) | ICD category: Bone | Connective and other soft tissue | Urinary organs* | Eye | Brain | Other parts of nervous system | Lymphoid tissue | Leukaemia | Other conditions in ICD, Ch. II | Conditions in MOTNAC but not in ICD, Ch. II | Total |
|---|---|---|---|---|---|---|---|---|---|---|---|
| Muscular and connective tissue | 3 | 69 | — | — | 1 | 2 | — | — | 43 | — | 118 |
| Complex mixed and stromal | — | — | 84 | — | — | — | — | — | 11 | — | 95 |
| Germ cell and teratomatous | — | 1 | — | — | 4 | 1 | — | — | 55 | — | 61 |
| Bone | 80 | — | — | — | — | — | — | — | — | — | 80 |
| Gliomatous | — | — | — | — | 263 | 24 | — | — | — | — | 287 |
| Neuroepitheliomatous | — | — | — | 52 | — | 114 | — | — | — | — | 166 |
| Leukaemic | — | — | — | — | — | — | 11 | 455 | — | — | 466 |
| Other lymphoid and haematopoietic | — | — | — | — | — | — | 100 | — | — | — | 100 |
| Other types in MOTNAC | 8 | 20 | 5 | 1 | 13 | 17 | — | — | 95 | 19 | 178 |
| Fibromata of bone (in ICD, Ch. II but not in MOTNAC) | 6 | — | — | — | — | — | — | — | — | — | 6 |
| Total | 97 | 90 | 89 | 53 | 281 | 158 | 111 | 455 | 204 | 19 | 1557 |

* Excluding urinary bladder.

retinoblastomata and neuroblastomata respectively (both classified as neuroepitheliomatous). The lymphoid and haematopoietic tissue groups defined by the 2 classifications were the same except for 11 cases of leucosarcoma (listed among the lymphomata in ICD and among the leukaemias in MOTNAC).

*Completeness of ascertainment*

The completeness of the notifications of cases reaching the CTR direct from hospital consultants and *via* the RCR (the latter including all cases identifiable from death registrations) could only be compared in respect of patients first seen in the period 1971–73, since it had not been recorded whether those seen earlier had been notified in both ways independently. HAA data were only available for the 1972–73 period.

The numbers of 1971–73 cases ascertained from the sources available throughout this period (other than cases notified to the CTR but neither accepted to be neoplastic nor known to the RCR) are shown in Table IV. These cases included 339 that were eligible for NCRS registration with the RCR, and 48 that were not. Of the 339, 94% (including 49% for whom

TABLE IV.—*Numbers of Conditions Diagnosed in Children in* 1971–73 *which were Registered with the Regional Cancer Registry under the National Cancer Registration Scheme and/or Notified Direct to the CTR*

| Registration particulars | Neoplasms of lymphoid and haematopoietic tissue | Other conditions | Total |
|---|---|---|---|
| Neoplasms eligible for NCRS registration | | | |
| Registered with RCR and notified direct to CTR: | | | |
| Death registration received | 75 | 81 | 156 |
| Death registration not received | 76 | 68 | 144 |
| Registered with RCR but not notified direct to CTR: | | | |
| Death registration received | 6 | 3 | 9 |
| Death registration not received | 3 | 8 | 11 |
| Not registered with RCR but notified direct to CTR | 5 | 14 | 19 |
| Total | 165 | 174 | 339 |
| Conditions not eligible for NCRS registration | | | |
| Registered with RCR but not notified direct to CTR: | | | |
| Death registration received | 1 | 2 | 3 |
| Death registration not received | 1 | 16 | 17 |
| Not registered with RCR but notified direct to CTR (valid CTR cases only) | 0 | 28 | 28 |
| Total | 2 | 46 | 48 |

TABLE V. *Children with Neoplasms in CTR Series Diagnosed in* 1972–73 (*Excluding Those Not Registrable Under the NCRS*) *Classified according to whether Records of Their Admission to Hospitals with any Neoplasms were found in Hospital Activity Analysis Files for January* 1972–*March* 1974

| Hospital Activity Analysis findings | Neoplasms of lymphoid and haematopoietic tissue | Other neoplasms | Total |
|---|---|---|---|
| Neoplasms notified to RCR and/or to CTR direct: | | | |
| Spell in registering hospital eligible for HAA: | | | |
| In HAA file of registering hospital | 97 | 101 | 198 |
| In HAA file of other hospital only | 2 | 6 | 8 |
| Not found in any HAA file | 5 | 8 | 13 |
| Care by registering agency not eligible for HAA: | | | |
| In HAA file of other hospital | 4 | 1 | 5 |
| Not found in any HAA file | 3 | 7 | 10 |
| Neoplasms not notified to RCR or to CTR direct: | | | |
| Found in HAA file | 0 | 3 | 3 |
| Total | 111 | 126 | 237 |

death registration particulars had been received) had been so registered and 94% had been notified direct to the CTR. The 48 cases ineligible for registration with the RCR comprised 28 that had been notified to the CTR alone with benign tumours, and 20 registered under the NCRS but found on further enquiry to be of benign neoplasms (6) or non-neoplastic conditions (10) or to involve non-residents of the study area (4).

The completeness of the HAA data for all known 1972–73 cases that were eligible for NCRS registration is examined in Table V. From a total of 237 cases 219 had been notified to the RCR or CTR by a hospital where they had received inpatient treatment for which there should have been an HAA record, but in 21 of the 219 this record was not found. Apart from these 219 cases there were 15 whose care by the notifying agency was outside the scope of HAA (5 inpatients of hospitals outside the Regional Health Authority's HAA catchment area, 8 outpatients, 1 nursing home case, and 1 general practitioner's) and 3 who were included in HAA without having been notified to the RCR or CTR. The total number of cases for whom HAA records were found was 214 (90%), including 13 cases in which the hospital(s) where the HAA records originated did not include the source of the RCR or CTR notification.

The amount of overlap between all the sources from which the 1972–73 cases were ascertained is examined in more detail in Tables VI and VII and the Figure. Table VI shows the distribution obtained when the 237 1972–73 cases were split simultaneously into those found and not found in the HAA file (shown in different columns) and into those notified and not notified to the RCR and/or direct to the CTR (shown on different rows as in Table IV). Table VII may be considered in 3 parts. In the first, we have used the data for 1971–73 in Table IV (supplemented by the percentage of cases ascertained from HAA alone in 1972–73—1·3%) to estimate the percentages of cases that might be ascertained from each source and combination of sources other than HAA and from none (column *b*). Secondly we have used data from Table VI to estimate what proportion of the cases in each of these ascertainment categories might have been ascertained using HAA data alone (column *e*). Thirdly, in columns *f* and *g* respectively, we have multiplied each of the percentages in column *b* by the corresponding proportion in column *e* and by the difference between the latter proportion and unity, to obtain estimates of the percentages of *all* cases that might be ascertained from every possible combination of the sources of data used. These percentages are also given in the

TABLE VI.—*Numbers of Neoplasms in CTR Series Diagnosed in* 1972–73 (*Excluding those not Registrable under the NCRS*) *by Source of Ascertainment*

| Registration particulars | Neoplasms of lymphoid and haematopoietic tissue: In HAA file | Neoplasms of lymphoid and haematopoietic tissue: Not in HAA file | Other neoplasms: In HAA file | Other neoplasms: Not in HAA file | Total: In HAA file | Total: Not in HAA file |
|---|---|---|---|---|---|---|
| Registered with RCR and notified direct to CTR | | | | | | |
| Death registration received | 46 | 2 | 45 | 7* | 91 | 9* |
| Death registration not received | 52 | 2 | 45 | 3 | 97 | 5 |
| Registered with RCR but not notified direct to CTR | | | | | | |
| Death registration received | 3 | 2 | 2 | 0 | 5 | 2 |
| Death registration not received | 2 | 0 | 5 | 2* | 7 | 2* |
| Not registered with RCR but notified direct to CTR | 0 | 2 | 11 | 3 | 11 | 5 |
| Not registered with RCR nor notified direct to CTR | 0 | – | 3 | – | 3 | – |
| Total | 103 | 8 | 111 | 15* | 214 | 23* |

* Including two NCRS registrations (one also notified direct to CTR and the other not) of cases seen at local NHS hospitals not participating in HAA.

TABLE VII.—*The Proportions of Childhood Neoplasms of Types Registrable under the National Cancer Registration Scheme that would be Ascertained from Each Source, given the Participation of All Local NHS Hospitals in HAA, the* 1972–73 *experience of HAA Recording, and the 1971–73 Experience of Notification Direct to the CTR and to the Regional Cancer Registry*

| Registration particulars | 1971–73 diagnoses: Total no. (*a*) | 1971–73 diagnoses: Percentage distribution* $\left(b=\frac{a(100-1\cdot3)}{\Sigma a}\right)$ | 1972–73 diagnoses†: Total no. (*c*) | 1972–73 diagnoses†: No. in HAA file (*d*) | 1972–73 diagnoses†: Proportion in HAA file $\left(e=\frac{d}{c}\right)$ | Expected percentage distribution of all cases: In HAA file ($f=be$) | Expected percentage distribution of all cases: Not in HAA file ($g=b(1-e)$) |
|---|---|---|---|---|---|---|---|
| Registered with RCR and notified direct to CTR | | | | | | | |
| Death registration received | 156 | 45·4 | 99 | 91 | 0·92 | 41·8 | 3·6 |
| Death registration not received | 144 | 41·9 | 102 | 97 | 0·95 | 39·8 | 2·1 |
| Registered with RCR but not notified direct to CTR | | | | | | | |
| Death registration received | 9 | 2·6 | 7 | 5 | 0·71 | 1·8 | 0·8 |
| Death registration not received | 11 | 3·2 | 8 | 7 | 0·88 | 2·8 | 0·4 |
| Not registered with RCR but notified direct to CTR | 19 | 5·5 | 16 | 11 | 0·69 | 3·8 | 1·7 |
| Not registered with RCR nor notified direct to CTR | — | 1·3 | 3 | 3 | 1·00 | 1·3 | 0·0 |
| Total | 339 | 100 | 235 | 214 | 0·91 | 91·3 | 8·6 |

* Calculated as if 1·3% of cases had been ascertained from HAA and from no other source in 1971 as well as in 1972–73.

† Excluding 2 cases seen only at local NHS hospitals not participating in HAA.

FIG.—Percentages of childhood neoplasms (of types registrable under the National Cancer Registration Scheme) that might be ascertained from different sources (from Table VII).

Figure. They confirm that for completeness of ascertainment there was little to choose between HAA records (which included 91% of known cases), RCR registrations (93%), and notifications to the CTR from consultants (93%). Between 98% and 99% of the cases could have been ascertained given any two of these three sources, and more than 95% if merely the sources not special to neoplasms (death registration and HAA) had been available.

*Diagnostic accuracy of cancer registry data*

To assess the accuracy of data collected under the NCRS, the morphology code numbers entered in NCRS files were compared with the final CTR diagnoses for all cases in the CTR series which had been registered under the NCRS for 1963–68. Morphological type was examined in preference to ICD category because in childhood neoplasms it is the more informative. 1963 was the first year for which the NCRS morphology data were sufficiently detailed and 1968 was the last for which all cases had been assigned a final CTR diagnosis at the time of the enquiry.

In about 9% of the cases, the morphological type in NCRS records differed enough from the final CTR diagnosis to fall into a different group of the simplified morphological classification used in Table II, but in more than half these cases the difference was only between a more and a less precise diagnosis or between leukaemia and another lymphoreticular neoplasm (Table VIII). The effect which these discrepancies had on the overall distribution of cases between categories of the simplified classification is shown in more detail in Table IX, which gives the additions and subtractions needed to convert the NCRS distribution to what was correct according to the CTR.

In addition to these errors, there were

TABLE VIII.—*Neoplasms in the CTR Series that were Registered under the National Cancer Registration Scheme in* 1963–68, *Classified According to whether they were Assigned to Categories in the Same Morphological Groups* (*Table II*) *by Both Agencies*

| Consistency of sources | Number | Percentage |
|---|---|---|
| Placed in same group by CTR and NCRS | 480 | 90·7 |
| Type uncertain (in " other " group) in CTR; not in " other " group in NCRS | 10 | 1·9 |
| Not in " other " group in CTR; type uncertain (in " other " group) in NCRS | 8 | 1·5 |
| " Leukaemic " in CTR; " other lymphoid and haematopoietic " in NCRS | 6 | 1·1 |
| " Other lymphoid and haematopoietic " in CTR; " leukaemic " in NCRS | 3 | 0·6 |
| Embryonal myosarcoma (in " muscular and connective tissue " group) in CTR; specific types in other groups in NCRS | 7 | 1·3 |
| Other discrepancies | 15 | 2·8 |
| Total | 529 | 100 |

TABLE IX.—*Morphological Distribution of Neoplasms in the CTR Series that were Registered under the National Cancer Registration Scheme in* 1963–68

| | Distribution by group according to NCRS | | | | Distribution by group according to CTR | | |
|---|---|---|---|---|---|---|---|
| Morphological group (MOTNAC) | Total | | NCRS group incorrect | | NCRS group incorrect | | Total |
| Muscular and connective tissue | 34 | – | 3 | + | 9 | = | 40 |
| Complex mixed and stromal | 30 | – | 2 | + | 2 | = | 30 |
| Germ cell and teratomatous | 7 | – | 1 | + | 1 | = | 7 |
| Bone | 16 | – | 2 | + | 1 | = | 15 |
| Gliomatous | 114 | – | 0 | + | 8 | = | 122 |
| Neuroepitheliomatous | 48 | – | 5 | + | 2 | = | 45 |
| Leukaemic | 178 | – | 4 | + | 7 | = | 181 |
| Other lymphoid and haematopoietic | 57 | – | 15 | + | 4 | = | 46 |
| Other | 45 | – | 17 | + | 15 | = | 43 |
| Total | 529 | – | 49 | + | 49 | = | 529 |

TABLE X.—*Morphological Distribution of Leukaemia Cases in the CTR Series that were Registered as Leukaemia by the National Cancer Registration Scheme in* 1963–68

| Morphological group (MOTNAC) | | Distribution by type according to NCRS | | | | Distribution by type according to CTR | | |
|---|---|---|---|---|---|---|---|---|
| Code number | Description | Total | | NCRS type incorrect | | NCRS type incorrect | | Total |
| 980 | Leukaemia, NOS | 33 | – | 8 | + | 38 | = | 63 |
| 982 | Lymphocytic leukaemia | 99 | – | 28 | + | 10 | = | 81 |
| 984 | Erythroleukaemia | 1 | – | 1 | + | 0 | = | 0 |
| 986 | Granulocytic leukaemia | 37 | – | 15 | + | 3 | = | 25 |
| 989 | Monocytic leukaemia | 4 | – | 0 | + | 1 | = | 5 |
| Total | | 174 | – | 52 | + | 52 | = | 174 |

of course cases in which the morphological type given in NCRS records differed in detail from the final diagnosis but was in the same group of the simplified classification. As an example of discrepancies of this kind, Table X shows that in 30% of the 174 cases diagnosed as leukaemia by both sources in Table IX, the cell type in the final CTR diagnosis differed from that recorded under the NCRS.

*Diagnostic accuracy of Hospital Activity Analysis data*

The only diagnostic data given in HAA records are ICD code numbers, which we have therefore compared with the final CTR diagnoses (coded in the same way) for all the 214 cases registrable under the NCRS for which HAA data were available. In 50 (23%) of these cases, the 3-digit code number given for HAA was not fully appropriate to the final diagnosis (Table XI). The discrepancies resembled those found when the NCRS diagnoses were analysed (Table VIII) in that more than half involved cases in which one diagnosis was merely more precise than the other, or in which the problem was one of dis-

TABLE XI.—*Neoplasms in the CTR Series for 1972–73 that were Also Found in Hospital Activity Analysis Files, Classified According to whether they were Assigned to the Same Three-digit Diagnostic Categories of the International Classification of Disease by Both Agencies*

| Consistency of sources | Number | Percentage |
|---|---|---|
| Placed in same category by CTR and HAA | 164 | 76·6 |
| Lymphosarcoma (200) in one source; lymphatic leukaemia (204) in other | 4 | 1·9 |
| Leukaemia specified as lymphoid (204) or myeloid (205) in one source but of undefined cell type (207) in other | 11 | 5·1 |
| Neoplasms of brain (191) in one source; of central nervous system not further defined (192) in other | 12 | 5·6 |
| Neoplasm assigned to same site in both sources, but malignant (140–199) in one and benign or unspecified (210–239) in other | 9 | 4·2 |
| Other discrepancies | 14 | 6·5 |
| Total | 214 | 100 |

TABLE XII.—*Distribution by Category (Based on the International Classification of Disease) of Neoplasms in the CTR Series for 1972–73 that were Also Found in Hospital Activity Analysis Files*

| | Distribution by category according to HAA | | | Distribution by category according to CTR | | | |
|---|---|---|---|---|---|---|---|
| Category of neoplasm (ICD) | Total | | HAA group incorrect | | HAA group incorrect | | Total |
| Malignant neoplasms | | | | | | | |
| Bone | 12 | − | 3 | + | 0 | = | 9 |
| Connective and other soft tissue | 4 | − | 3 | + | 3 | = | 4 |
| Urinary organs (excluding bladder) | 14 | − | 0 | + | 0 | = | 14 |
| Eye | 3 | − | 1 | + | 0 | = | 2 |
| Brain | 28 | − | 1 | + | 15 | = | 42 |
| Other parts of nervous system | 23 | − | 12 | + | 1 | = | 12 |
| Lymphoid tissue | 21 | − | 4 | + | 3 | = | 20 |
| Leukaemia | 80 | − | 1 | + | 4 | = | 83 |
| Other | 21 | − | 7 | + | 8 | = | 22 |
| Benign or unspecified neoplasms | | | | | | | |
| Nervous system | 4 | − | 3 | + | 1 | = | 2 |
| Other | 4 | − | 3 | + | 3 | = | 4 |
| Total | 214 | − | 38 | + | 38 | = | 214 |

tinguishing between different lymphoid and haematopoietic neoplasms.

In 38 of the 50 discrepancies, the HAA and CTR diagnoses were in different categories of the short classification (based on the ICD) that we used in Table I. The distribution of these cases is examined in Table XII.

### DISCUSSION

### *Classification*

Our trial of the ICD and the Morphology Section of MOTNAC leads us to prefer the latter for studies of the epidemiology and pathology of children's tumours. The ICD splits neoplasms primarily into malignant, benign, and of unspecified malignancy. A few of the categories in these main groups relate to specific tissues (e.g. lipoma, malignant melanoma) but most to specific anatomical sites. This defining of most cancers in terms of site fits the ICD for use where histological data are not available. Its use in aetiological studies of adult cancers can also be justified on the grounds that at almost every site that is commonly affected in this age group the vast majority of cases are of carcinoma, and that locally acting extrinsic factors which differ very much from site to site probably play a major part in the aetiology of these cases. Most childhood neoplasms however arise at sites less likely to be affected by localized extrinsic factors; and the cancers that occur relatively frequently in children at some of these sites (or at sites that are grouped together by the ICD) are of more than one type. For example, Ewing's tumour and osteosarcoma are both cancers of bone (ICD category no. 170). Similarly, neuroblastomata of sympathetic ganglia are assigned on the basis of site to the same 3-digit category as meningeal and spinal cord malignancies (" 192: malignant neoplasm of other parts of nervous system ") in the current ICD (the 8th Revision), and have recently been grouped with soft tissue sarcomas under " 171: malignant neoplasm of connective and other soft tissue " by the International Conference for the 9th Revision of the ICD.

The Morphology Section of MOTNAC classifies primarily by histogenesis, and subdivides the groups so defined into malignant, benign, and of doubtful malignancy. It includes separate categories for all the tumour types mentioned in the last paragraph (even though for brevity they have been grouped with others in the abbreviated form of this classification used above—e.g. in Table II), and our use of it to classify childhood neoplasms raised no major problems. We have a few reservations about its nomenclature (e.g. its use of the term " lymphocytic leukaemia " for lymphoid leukaemia—which in children is not generally lymphocytic but lymphoblastic); but a revised and expanded version in which some of these anomalies (including the example quoted) have been corrected has recently been prepared, and is now undergoing field trials as part of an " International Classification of Diseases for Oncology " which it is intended to publish as a supplement to the ICD (9th Revision). The contents of the MOTNAC-based categories listed in Tables II and IX would have been the same if the revised Morphology Section had been used, except that bone fibromata and cases of Letterer-Siwe's disease would have been included (under " bone " and " other lymphoid and haematopoietic " respectively).

As the revised Morphology Section has all the merits of the original, and is in addition likely to become the standard classification based on histogenesis for neoplasms at all ages, we recommend that this should be the main classification used in studies of children's tumour pathology and epidemiology whenever possible, and that any demands for a more detailed classification should in general be met by subdividing existing categories rather than by developing a whole new system. Bearing in mind, however, that for most communities the only available data relate to cases analysed by ICD category, we consider that, to facilitate international comparisons, the distribution of cases by ICD category as well as by morphology should be given in reports on the frequency of childhood neoplasms in large series. Although the ICD discriminates less well than the Morphology Section of MOTNAC between the commoner types of childhood neoplasms, several of these (notably Wilms' tumour, retinoblastoma, and the lymphoid and haematopoietic neoplasms) are practically specific to ICD categories which include hardly any childhood cases of other neoplasms (Table III). One might therefore hope to detect any marked variations in the frequency of these tumour types by comparing the incidence of or mortality from neoplasms of the related sites.

*Completeness of ascertainment*

The distribution of our cases between sources (Tables VI and VII and Figure) suggests that practically all children who presented with life-threatening neoplasms in the period 1972–73 were ascertained. Even in HAA records, the least productive of our 3 main sources, 91% of the 1972–73 cases of neoplasms registrable under the NCRS and known to any of the 3 sources were identified. A further 7% are found when HAA and NCRS records (the least productive pair of sources) are both used, and addition of the third source (notifications to the CTR from clinicians) only contributes 2% more. The latter figure is not quite as low as one would expect it to be if the first 2 sources were independent (in which case only 0·5%* of all 1972–73 cases would be expected to be missed by both); in other words, cases notified to one of these agencies appear—not surprisingly—to be more likely than other cases to be notified to the other agency also, and in these circumstances the level of ascertainment achieved using data from both is likely to be lower than if the agencies were independent. Nevertheless, the diminishing returns seen when one source is supplemented by a second and then by a third strongly suggest that if more sources still were to be added, the yield of further cases would be negligible.

If our inference that virtually all severe cases diagnosed in the period 1972–73 were ascertained is correct, we can use the proportions of cases in this series that were ascertained from routine sources as estimates of the proportions of all severe cases that might be so ascertained. These estimates amount to more than 98% for ascertainment from cancer and death registrations and HAA records, and more than 95% if cancer registration data for survivors are excluded. From the first of these figures we conclude that in areas where all hospitals participate in both HAA and NCRS (or in similar schemes), a high enough level of ascertainment of children's cancer for most epidemiological purposes may be achieved by considering merely children who had neoplasms according to either or both of these sources.

The estimate that ascertainment from death registrations and HAA would be 95% complete suggests that the ending of cancer registration as a separate hospital activity—which has already happened in Wales so far as inpatients are concerned, following the introduction of HAA (West, 1973)—would make little difference to the completeness of ascertainment that is possible from routine sources. The completeness of one of the two remaining sources of this kind—death registration—is of course being eroded by advances in treatment, but there is scope for the other—HAA—to rise above its level for 1972–73, when nearly 10% of the patients notified to the RCR or CTR from HAA hospitals to which they had been admitted had no HAA record of admission to these hospitals with neoplasms (Table V). Omissions of this kind should become rarer as the hospitals to which HAA was new in 1972 acquire more experience of the system, and it is also to be hoped that HAA will eventually be extended to cover outpatient as well as inpatient episodes, and to include all hospitals in the country in one standardized system, the benefits of which would include access by each Health Authority to data on members of its population who are treated elsewhere. If HAA had had these features in 1972–73, all but 2 of the cases undergoing "care by registering agency not eligible for HAA" (Table V) might have been included (the exceptions being the nursing home's case and the general practitioner's), which would have added more than 3% to the proportion of all registrable children's neoplasms ascertained from HAA. Given these improvements, it should therefore be possible to identify a high enough proportion of children with cancer for most epidemiological purposes, merely by scanning HAA and death records.

*Accuracy of ascertainment*

Although almost all cases of childhood cancer can apparently be identified from routine sources, our findings strongly suggest that the diagnostic data available from these sources are inadequate. From the expert review of all the available clinical and pathological findings carried out in every case for the CTR, it seems that nearly 6% of the cases registered under the NCRS in 1971–73 were not eligible for

* $\left[\frac{ab}{c} \div \left(a+b+c+\frac{ab}{c}\right)\right]$, where $a$ and $b$ are the numbers of cases found in the records of HAA but not NCRS and of NCRS but not HAA respectively, and $c$ is the number common to both sources.

this registration (Table IV); that in nearly one fifth of the eligible cases registered in 1963–68, the NCRS coding and the final CTR diagnosis in terms of morphology either fell into different categories of our simplified classification (Tables VIII and IX) or at least—in cases agreed to have leukaemia—differed in cell type (Table X); and that nearly a quarter of the eligible cases first seen in 1972–73 for whom entries were found in HAA files had not been assigned there to the 3-digit ICD categories most appropriate to them (Tables XI and XII). Admittedly, some of these discrepancies may be regarded as trivial; some may have been due to inexperience, since the 1963–68 and 1972–73 data were collected when cancer registration and HAA respectively were relatively new to the hospitals concerned; and in some cases there may have been room for more than one opinion as to the correct diagnosis. Nevertheless, the magnitude of the discrepancies forces us to conclude that although purely routine records may be used to *identify* children with cancer, the diagnoses they record should not be accepted unquestioningly in any epidemiological study of childhood neoplasia, local or national. Instead, they should always be reviewed by experts in the light of the case notes and (if possible) histological preparations. To promote consistency in this procedure, the reviewers should if possible be the same throughout the survey, since there are some issues—e.g. whether certain cases of leukaemia are of lymphoid or undefined (blast cell) type —on which even expert observers not infrequently differ. As well as yielding useful epidemiological information, a register of childhood neoplasms compiled in this way can provide an excellent base for clinical and laboratory research.

This work was done as part of an epidemiological study of children's tumours supported by United States Public Health Service Research Grant No. CA14992 from the National Cancer Institute. We are also deeply indebted to the DHSS Childhood Cancer Research Group at Oxford, to the staff of the Office of Population Censuses and Surveys and of the North Western Regional Health Authority, and to hospital consultants and Medical Records Officers throughout the study area, all of whom have been most helpful in making data available to us; to the pathologists who provided histological reports on the cases studied; and to Mrs Cora Christmas and Miss Catherine Hall who helped in analysing the data.

### REFERENCES

ALDERSON, M. R. & MEADE, T. W. (1967) Accuracy of Diagnosis on Death Certificates Compared with that in Hospital Records. *Br. J. prev. soc. Med.*, **21**, 22.

HANAWA, Y. (1975) Children's Cancer in Japan by Registration. In: *All Japan Children's Cancer Registration* 1969–73. Tokyo: Children's Cancer Association of Japan. p. 31.

HEASMAN, M. A. & LIPWORTH, L. (1966) *Accuracy of certification of cause of death* (*Studies on Medical and Population Studies*, No. 20). London: HMSO.

HINDS, S. W., WILSON, L. M. K. & DRAPER, G. J. (1974) Childhood Malignant Disease in Britain 1953–71. *Hlth Social Service J.*, **84**, 880.

MARSDEN, H. B. & STEWARD, J. K. (1968) (Eds.) *Tumours in Children* (*Recent Results in Cancer Research*, **13**). Berlin: Springer.

MILLER, R. W. (1969) Childhood Cancer and Congenital Defects: A Study of U.S. Death Certificates During the Period 1960–66. *Pediat. Res.*, **3**, 389.

MILLER, R. W. (1971) Deaths from Childhood Leukemia and Solid Tumors among Twins and Other Sibs in the United States, 1960–67. *J. natn. Cancer Inst.*, **46**, 203.

PERCY, C. L., BERG, J. W. & THOMAS, L. B. (1968) (Eds.) *Manual of Tumor Nomenclature and Coding: 1968 Edition.* American Cancer Society Inc.

STEWART, A. M., WEBB, J. W. & HEWITT, D. (1958) A Survey of Childhood Malignancies. *Br. med. J.*, i, 1495.

WEST, R. R. (1973) Cancer Registration by means of Hospital Activities Analysis. *Hospital and Health Services Review*, 372.

YOUNG, J. L. & MILLER, R. W. (1975) Incidence of Malignant Tumors in U.S. Children. *J. Pediatr.*, **86**, 254.